# Performance of a seed amplification assay for misfolded alpha-synuclein in cerebrospinal fluid and brain tissue in relation to Lewy body disease stage and pathology burden

**DOI:** 10.1007/s00401-023-02663-0

**Published:** 2024-01-19

**Authors:** Giuseppe Mario Bentivenga, Angela Mammana, Simone Baiardi, Marcello Rossi, Alice Ticca, Franco Magliocchetti, Andrea Mastrangelo, Anna Poleggi, Anna Ladogana, Sabina Capellari, Piero Parchi

**Affiliations:** 1 Department of Biomedical and Neuromotor Sciences, University of Bologna, Bologna, Italy; 2 IRCCS, Istituto Delle Scienze Neurologiche di Bologna, Bologna, Italy; 3 Department of Neurosciences, Istituto Superiore di Sanità, Rome, Italy

**Keywords:** RT-QuIC, α-Synuclein, Lewy bodies, Parkinson’s disease, Diagnosis, Amygdala

## Abstract

The development of in vitro seed amplification assays (SAA) detecting misfolded alpha-synuclein (αSyn) in cerebrospinal fluid (CSF) and other tissues has provided a pathology-specific biomarker for Lewy body disease (LBD). However, αSyn SAA diagnostic performance in early pathological stages or low Lewy body (LB) pathology load has only been assessed in small cohorts. Moreover, the relationship between SAA kinetic parameters, the number of αSyn brain seeds and the LB pathology burden assessed by immunohistochemistry has never been systematically investigated. We tested 269 antemortem CSF samples and 138 serially diluted brain homogenates from patients with and without neuropathological evidence of LBD in different stages by the αSyn Real-Time Quaking-Induced Conversion (RT-QuIC) SAA. Moreover, we looked for LB pathology by αSyn immunohistochemistry in a consecutive series of 604 Creutzfeldt–Jakob disease (CJD)-affected brains. αSyn CSF RT-QuIC showed 100% sensitivity in detecting LBD in limbic and neocortical stages. The assay sensitivity was significantly lower in patients in early stages (37.5% in Braak 1 and 2, 73.3% in Braak 3) or with focal pathology (50% in amygdala-predominant). The average number of CSF RT-QuIC positive replicates significantly correlated with LBD stage. Brain homogenate RT-QuIC showed higher sensitivity than immunohistochemistry for the detection of misfolded αSyn. In the latter, the kinetic parameter lag phase (time to reach the positive threshold) strongly correlated with the αSyn seed concentration in serial dilution experiments. Finally, incidental LBD prevalence was 8% in the CJD cohort. The present results indicate that (a) CSF RT-QuIC has high specificity and sufficient sensitivity to detect all patients with LB pathology at Braak stages > 3 and most of those at stage 3; (b) brain deposition of misfolded αSyn precedes the formation of LB and Lewy neurites; (c) αSyn SAA provides “quantitative” information regarding the LB pathology burden, with the lag phase and the number of positive replicates being the most promising variables to be used in the clinical setting.

**Supplementary Information:**

The online version contains supplementary material available at 10.1007/s00401-023-02663-0.

## Introduction

Lewy body disease (LBD), the second most prevalent neurodegenerative disorder after Alzheimer's disease (AD), is characterized by the misfolding and aggregation of alpha-synuclein (αSyn), resulting in the formation of cytoplasmatic neuronal inclusion in cell bodies (i.e., Lewy bodies [LBs]) and nerve terminals (i.e., Lewy neurites [LNs]) [36, 61]. Besides being the pathological hallmark of dementia with Lewy bodies (DLB) and the majority of Parkinson’s disease (PD) cases, LB pathology often manifests as co-pathology in patients with AD, and it also affects a variable proportion of aged individuals lacking clinical signs of motor disturbances or cognitive decline. The latter condition is known as “incidental LBD” (iLBD) and likely represents the pathological preclinical stage of PD or DLB [22, 33, 35]. As it is increasingly recognized for misfolded proteins linked to neurodegeneration, misfolded αSyn can act as a seed to induce the conversion of the normal cellular αSyn, allowing for the transcellular propagation of LB pathology in a prion-like manner [24, 67]. In the CNS, LBD most often starts in the medulla oblongata and then spreads intracerebrally, following an ascending course from the brainstem to the cerebral cortex, passing through the limbic areas [12, 13, 28, 63]. Accordingly, three major disease stages have been identified, i.e., the brainstem (I), limbic or transitional (II), and neocortical (III) [37, 43]. A second, more detailed staging scheme, proposed by Heiko Braak [12, 13], recognizes six stages, among which the first three, corresponding to the brainstem stage, define in more detail the progressive involvement of the brainstem from the medulla oblongata to the pons and the midbrain reaching the basal forebrain and the hypothalamus before involving the limbic areas. There are well-established exceptions to the LB pathology progression described above [2, 50, 69], the most common being the amygdala-predominant variant, in which a widespread αSyn deposition is found in the amygdala, associated with only mild and focal LB pathology in the brainstem, not precisely following the Braak staging, often in association with moderate or severe AD pathology [19, 29, 40, 41, 59, 63, 65].

The development of in vitro seed amplification assays (SAA) that indirectly detect misfolded αSyn in the cerebrospinal fluid (CSF) and other tissues has recently provided a pathology-specific biomarker for LBD, thus representing a breakthrough in the field [14]. However, only a few studies in limited cohorts have correlated the in vivo outcome based on αSyn SAA result against the gold standard of the postmortem LB pathology assessment [3, 7, 10, 21, 27, 55]. At the same time, none has, to date, specifically correlated the kinetic parameter values of αSyn SAA with the LB pathology burden.

In this study, we took advantage of a unique collection of CSF and brain pairs from the same patients collected within a limited time frame due to a rapidly progressive disease to address critical questions regarding the sensitivity of our αSyn SAA in detecting LB pathology at different stages and its overall specificity.

Moreover, to provide new insights into the relationship between the kinetic parameters of the αSyn SAA and the number of αSyn seeds, and into the correlation between LB pathology burden assessed by immunohistochemistry and the αSyn SAA outcome, we performed the αSyn SAA on tissue homogenates from different brain areas affected by LB pathology at different stages.

Finally, we used a cohort of consecutive well-characterized patients with Creutzfeldt–Jakob disease (CJD) lacking any clinical evidence of cognitive decline and/or motor disturbances before the rapid onset of symptoms related to CJD to estimate the frequency of iLBD pathology in the elderly population.

## Materials and methods

### Case selection

A series of 862 consecutive brains have been collected at the Neuropathology Laboratory (NP-Lab) at the Istituto delle Scienze Neurologiche di Bologna (ISNB) between 2003 and 2022. The cohort (NP cohort) mainly included cases of progressive dementia, often with a rapidly progressive course, or of atypical parkinsonism, primarily belonging to the following diagnostic categories: CJD, LBD, AD, frontotemporal lobar degeneration, multiple system atrophy (MSA), progressive supranuclear palsy, encephalitis, lymphoma, and vascular dementia. Neuropathological diagnosis of neurodegenerative diseases was made according to established criteria [2, 17, 18, 23, 38, 45, 48]. All brains have been classified into αSyn LB+ and αSyn LB− subgroups based on protein aggregate assessment by immunohistochemistry (see below for further details).

For the study's primary aims, we selected from the NP cohort all the participants with antemortem CSF available and those with a final CJD diagnosis. The first group comprised 269 patients (CSF cohort), including 210 subjects lacking LB pathology (αSyn LB− group) and 59 with LB pathology (αSyn LB+ group). In the αSyn LB− group, we excluded CSF samples for which the time between lumbar puncture (LP) and death was more than one year, while we included all available samples in the αSyn LB+ group (of note, only 7/59 had a time LP-death > 1 year). The second group included 604 brains with a CJD diagnosis (CJD cohort).

### Neuropathological studies

Neuropathological examination was performed at NP-Lab using standardized procedures as described [39]. Briefly, each brain was divided sagittally, and the left hemibrain fixed in 10% buffered formalin while the right one was sectioned coronally and then immediately frozen at − 80 °C in sealed plastic bags. The formalin-fixed left hemibrain was serially sectioned in 1 cm slices, and tissue blocks from 24 regions were processed routinely to obtain paraffin-embedded brain tissue blocks [49].

Seven μm thick sections from each block were stained with hematoxylin–eosin for screening. Also, immunohistochemistry with antibodies specific for αSyn (LB509, dilution 1:100, Thermo Fisher Scientific, and KM51, dilution 1:500, Novocastra), phospho-tau (*p*-tau) (AT8, dilution 1:100, Innogenetics), and Aβ (4G8, dilution 1:5000, Signet Labs), was applied to all cases using several brain regions, mainly following established consensus criteria [1, 2, 16, 45, 48]. An experienced neuropathologist (PP) formulated the final diagnosis, assigned the stage of LB pathology according to consensus criteria [2, 4], and classified each αSyn LB+ case according to the level of AD neuropathologic change (ABC score) [1, 45].

For the semiquantitative assessment of LB pathology, brains with αSyn positive LBs or LNs in at least one section and available CSF samples were examined independently by two evaluators (PP and GMB). Specifically, αSyn immunoreactivity was evaluated semi-quantitatively (0-no immunoreactivity; 1-mild; 2-moderate; 3-prominent immunoreactivity) in the following regions: medulla oblongata (vagus dorsomedial nucleus, intermediate reticular zone), pons (locus coeruleus, raphe), substantia nigra, hypothalamus, basal forebrain (Meynert's basal nucleus and surrounding gray matter), amygdala, hippocampus (CA2–CA4 sectors), parahippocampal gyrus, cingulate gyrus, middle temporal gyrus, middle frontal gyrus, occipital cortex, and cerebellum. We scored the neuronal LBs and LNs separately. Each case was given a combined “LB score” (range 0–78).

### Evaluation of αSyn seeding activity in CSF and brain homogenates by SAA

All CSF samples were obtained by LP at the L3/L4 or L4/L5 level following a standard procedure. They were subsequently divided into 400–500 μl aliquots and stored in polypropylene tubes at − 80 °C until analysis. We performed the CSF and brain homogenates αSyn Real-Time Quaking-Induced Conversion (RT-QuIC) SAA, including the purification of recombinant wild-type human αSyn, as previously described with minor analysis modifications [26, 53–55]. Samples and controls were deemed positive after the first run when at least three out of four replicates reached a threshold arbitrarily set at 30% of the median of the maximum fluorescence intensity (Imax) reached by the positive control (positive) replicates. To minimize the risk of false-positive results, we repeated three times the analysis of samples showing seeding activity in only one or two of four replicates in the first run. Then, we considered the result “positive” only when at least 4 of the 12 total replicates reached the threshold.

Tenfold serial dilutions were prepared for the αSyn seeds quantification in brain homogenates, and each sample was run in quadruplicates. The dilution series was analyzed until the αSyn RT-QuIC SAA yielded ≤ 2 out of 4 positive replicates. Then, we used the Spearman–Kärber algorithm, which has been previously applied for the quantification of prion seeding activity in analogy to a bioassay’s lethal dose (LD_50_) calculation [20, 30, 68], to estimate a seeding dose or dilution at which only 50% (e.g., 2 of 4) of replicates are positive (50% seeding doses or SD_50_). Next, to estimate the αSyn seed concentration in each brain area, a logSD_50_ per mg of tissue was calculated. Finally, we obtained a combined logSD_50_ score for each brain by summing all brain areas' logSD_50_/mg values to compare the overall αSyn seeding activity between brains.

We used eight batches of αSyn recombinant protein throughout the study for the brain homogenate analyses. Each batch underwent a quality control test before use. We ran at least one positive and negative control on each plate. The positive controls were chosen from patients with definite LBD whose brain homogenates or CSF samples gave four positive replicates during screening. In each validated experiment (plate) included in the final analysis, the positive controls showed at least three positive replicates. The average inter-batch coefficient of variation (CV) of the lag phase (i.e., the time required for the αSyn RT-QuIC SAA reaction to reach the above-mentioned “positivity” threshold) and the Imax in positive controls were 10.7% and 22.5%, respectively. All αSyn RT-QuIC SAA experiments were performed at the NP-Lab by personnel blinded to neuropathological diagnosis.

### Statistical analysis

αSyn RT-QuIC SAA relative fluorescence response data were analyzed and plotted using the software GraphPad Prism 8.3. The Imax, the lag phase and the area under curve (AUC) were extracted. The normality of the distribution of these variables from each group was assessed using the Kolmogorov–Smirnov test. The protein aggregation rate (PAR) was calculated as the inverse of the lag phase (1/lag phase). Mann–Whitney U test, one-way analysis of variance (followed by Tukey’s multiple comparisons post hoc test), and Kruskal–Wallis test (followed by Dunn’s post hoc analysis) were used to compare the continuous variables between the groups. The Spearman’s rank correlation coefficient was computed to determine significant associations between parameters. *P* values < 0.05 were considered statistically significant.

## Results

### Neuropathological analysis of LB pathology

The neuropathological analysis of the NP cohort revealed LB pathology in the brains of 114 (13.2%) patients. Forty-eight of 604 (7.9%) participants in the CJD group showed LB pathology, whereas 66 of 258 (25.6%) individuals had LB pathology in the non-CJD group.

Of the 114 patients with LB pathology in the NP cohort, 29 showed a neocortical stage, 37 showed a limbic stage, 42 a brainstem stage, and only 6 belonged to the amygdala-predominant variant. The relative proportion of subjects in each LBD stage of LB pathology in the whole cohort and the major subgroups is shown in Table 1.

**Table 1:** Demographic characteristics and neuropathological assessment results in the whole NP cohort and in the main diagnostic groups

Main NP-diagnosis	*N*	Age at death	*F* (%)	αSyn LB+				αSyn LB−
				Neocortical	Limbic	Brainstem	Amygdala-predominant	
Whole NP cohort	862	69.1 ± 9.8	49.4	29 (3.4%)	37 (4.3%)	42 (4.9%)	6 (0.7%)	748 (86.8%)
CJD	604	68.4 ± 9.4	50.0	2 (0.3%)	19 (3.1%)	27 (4.5%)	-	556 (92.1%)
Non-CJD^a^	258	71.0 ± 10.5	48.0	27 (10.5%)	18 (6.9%)	15 (5.8%)	6 (2.3%)	192 (74.4%)

Age at death is expressed as mean ± SD

*CJD*, Creutzfeldt–Jakob disease; *LB*, Lewy body pathology; *NP*, neuropathological

^a^main diagnostic groups: Alzheimer’s disease, Lewy body disease, subcortical arteriosclerotic encephalopathy

Rates of AD pathology were high across the cohort with LB pathology: 30 of 66 (45.5%) participants with limbic or neocortical LBD stages had intermediate or high degrees of AD neuropathological changes, as did almost all (5/6, 83.3%) cases with the amygdala-predominant variant.

### Sensitivity and specificity of the αSyn RT-QuIC SAA using CSF Samples

We performed the αSyn RT-QuIC SAA in 269 patients with antemortem CSF available, including 210 subjects lacking LB pathology and 59 with LB pathology assessed by αSyn immunostaining (see Supplementary Table 1 for further information about the pathological entities in the αSyn LB+ group). Demographic characteristics and αSyn RT-QuIC SAA results in the CSF cohort are shown in Table 2. The CSF αSyn RT-QuIC SAA yielded an overall specificity against the gold standard (i.e., neuropathologic examination) of 98.6%. Unexpected positive results were limited to three cases with a primary neuropathological diagnosis of Wernicke’s encephalopathy, AD, and sporadic CJD (sCJD), which showed no detectable αSyn brain deposits, despite the positivity by αSyn RT-QuIC SAA. Of note, the assay yielded negative results also in the two MSA cases.

**Table 2:** Demographic characteristics and CSF analysis results in the CSF cohort

	*N*	*F* (%)	Age at death (years)	Time LP-death (months)	N. αSyn RT-QuIC SAA-positive	N. αSyn RT-QuIC SAA negative	Sensitivity (%)	Specificity (%)
αSyn LB−	210	47.1	70.1 ± 9.4	1.8 ± 2.7	3	207		98.6
αSyn LB+	59	42.3	73.0 ± 7.9	5.0 ± 12.4	48	11	81.4	
McKeith stage								
Neocortical	15	53.3	77.5 ± 4.7	8.2 ± 12.2	15	0	100	
Limbic	17	47.0	72.8 ± 7.1	3.5 ± 11.9	17	0	100	
Brainstem	23	39.1	70.3 ± 8.5	1.9 ± 5.4	14	9	60.9	
Braak stage								
1	4	50.0	66.5 ± 5.9	0.5 ± 0.5	2	2	50.0	
2	4	50.0	69.7 ± 9.2	1.0 ± 0.8	1	3	25.0	
3	15	33.3	71.4 ± 9.0	2.5 ± 6.7	11	4	73.3	
4	5	60.0	73.6 ± 9.1	0.4 ± 0.5	5	0	100	
5	12	41.6	72.5 ± 6.6	4.9 ± 14.2	12	0	100	
6	15	53.3	77.5 ± 4.7	8.2 ± 12.2	15	0	100	
Amygdala-predominant	4	0	72.7 ± 11.9	16.7 ± 31.5	2	2	50	
LB score (0–78)					33.0 ± 15.4	7.0 ± 4.6		

Age at death, time LP-death, and LB score are expressed as mean ± SD

*LB*, Lewy body; *LP*, lumbar puncture

**Fig. 1:**
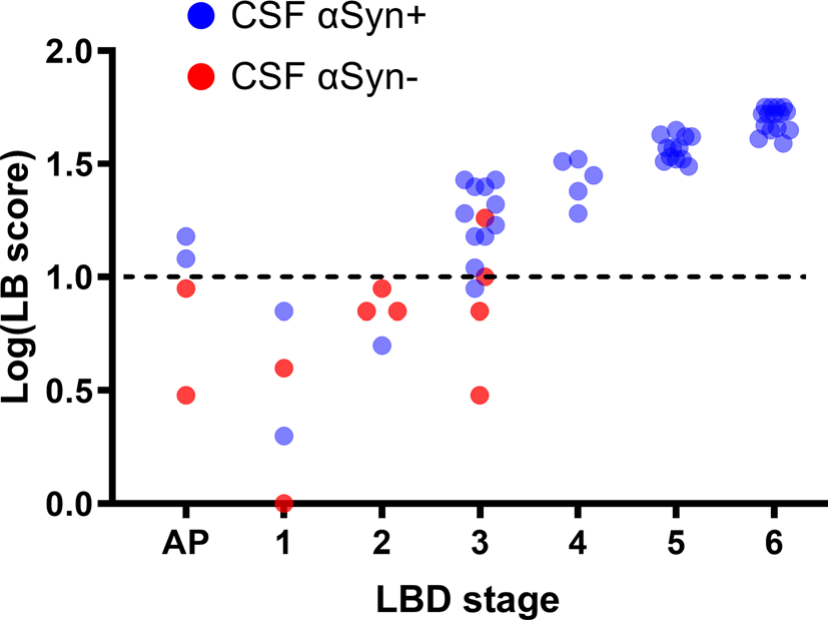
CSF αSyn RT-QuIC SAA results in αSyn LB+ patients according to Braak LBD stage and LB score. Red dots represent αSyn RT-QuIC SAA negative patients, while blue dots indicate the αSyn RT-QuIC SAA-positive ones. Almost all negative patients exhibited a low LBD (or amygdala-predominant) stage or low LB scores. *AP*, amygdala-predominant; *CSF*, cerebrospinal fluid; *LBD*, Lewy body disease

Of the 59 individuals with LB pathology, 48 tested positive by the αSyn RT-QuIC SAA, resulting in an assay sensitivity to detect αSyn pathology of 81.4% (Table 2). However, we observed significant differences in the test sensitivity after stratification by the extent and load of LB pathology. The αSyn RT-QuIC SAA had a sensitivity of 100% in detecting αSyn seeds in limbic and neocortical LBD stages (i.e., Braak stages > 3), 60.8% in the brainstem stage (73.3% and 37.5% in Braak stages 3 and 1/2, respectively) and 50% in detecting the amygdala-predominant variant. Interestingly, there was a clear association between the quantitative score of LB pathology and the result of the αSyn RT-QuIC SAA (Fig. 1). LB score in CSF αSyn + individuals was significantly higher than in CSF αSyn— ones (*p* < 0.0001) (Table 2). Indeed, virtually all (44/45) individuals with a score > 10 were positive, whereas most of those (10/14) with a score below this threshold were negative (Fig. 1).

Specifically, the “negative” group included two individuals showing only focal LBs and LNs in the medulla oblongata (Braak stage 1), three with a Braak stage 2 with sparse LBs and LNs in the medulla oblongata and pons, four in Braak stage 3 (among whom, one showed an atypical distribution with sparse LBs and LNs in the medulla oblongata and substantia nigra skipping the pons) and two with the amygdala-predominant variant showing moderate LB pathology in the amygdala associated with a mild pathology limited to the medulla oblongata.

Among the positive cases, we found a strong association between the number of positive replicates and the LB score (*r* = 0.7715, 95% confidence interval [CI] 0.6378 to 0.8601; *p* < 0.0001), and the Braak stage (*r* = 0.7554, 95% CI 0.6143–0.8497; *p* < 0.0001). Specifically, 80% and 20% of patients in neocortical stages showed 4/4 and 3/4 positive replicates, respectively. In the limbic stage, 47%, 35%, and 18% of cases had 4/4, 3/4, and 2/4 positive replicates, respectively. Among those in the brainstem stage, 7%, 42%, and 51% showed 4/4, 3/4, and 2/4 positive replicates, respectively. Finally, the two αSyn RT-QuIC SAA-positive amygdala-predominant cases showed 2/4 positive replicates (Supplementary Fig. 1).

### Detection of αSyn seeds in brain samples from αSyn LB+ and αSyn LB-/ CSF αSyn RT-QuIC SAA negative patients

Next, we analyzed a subset of 20 patients who had frozen brain tissue available, including 5 brainstem, 5 limbic, 3 neocortical, 3 amygdala-predominant, and 4 αsyn LB−/CSF αSyn RT-QuIC negative cases. Tenfold serial dilutions of brain homogenates were obtained from the medulla oblongata, substantia nigra, amygdala, basal forebrain, cingulate gyrus, temporal cortex, frontal cortex, cerebellum, and occipital cortex. The 4 αSyn LB− participants were negative by the αSyn RT-QuIC SAA in all examined brain regions, consistent with the results obtained with the antemortem CSF. The opposite was true for the limbic and neocortical cases, in which we detected αSyn seeding activity in all tested brain areas over a varying dilution range (from 10^–4^ to 10^–21^), depending on the case and the brain area. In contrast, participants showing the brainstem stage or the amygdala-predominant patients showed significant heterogeneity, mainly depending on the LB pathology load. Specifically, patients #51 (Braak 1) exhibited αSyn seeding activity only in the medulla oblongata (dilution range 10^–4^–10^–6^), patient #48 (Braak 3) was αSyn RT-QuIC SAA-positive in the medulla oblongata and the substantia nigra (dilution range 10^–4^–10^–13^), patient #50 (Braak 3) was αSyn RT-QuIC SAA-positive in brainstem areas, basal forebrain, amygdala, and cingulate gyrus (dilution range 10^–4^-10^–6^), and patients #43 and #54 (both Braak 3) showed αSyn seeds in all brain areas (dilution range of 10^–4^–10^–14^, with the highest dilutions needed in brainstem areas and basal forebrain). All three subjects with amygdala-predominant variant (#37, #41, and #57) were positive in the amygdala (dilution range 10^–4^–10^–14^), basal forebrain and medulla (dilution range 10^–4^–10^–7^). Moreover, αSyn seeding activity was variably detected in a few additional brain areas, such as the substantia nigra and cingulate gyrus (cases #37 and #41) or the temporal and occipital cortices (case #41) (dilution range 10^–4^–10^–6^).

Notably, 47 of the 138 brain samples showed αSyn seeding activity despite the negative immunohistochemical assessment, highlighting the increased sensitivity of the αSyn RT-QuIC SAA for the detection of misfolded αSyn compared to the standard immunohistochemical approach.

**Fig. 2:**
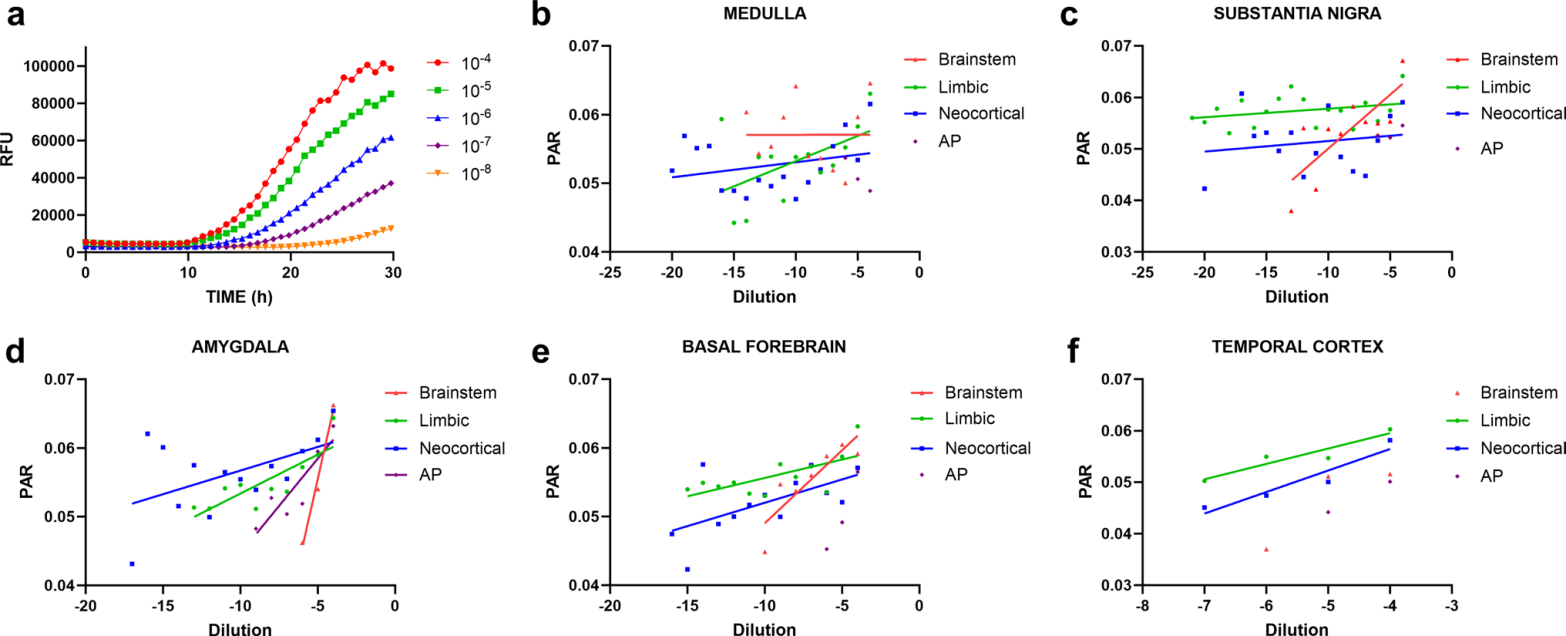
αSyn RT-QuIC SAA results in brain homogenates from αSyn LB+ patients. **a** Progressive reduction of seeding activity in brain homogenates of cingulate gyrus from case #24 along the dilution series. Each curve represents the average RFU values of positive quadruplicates. Standard deviation (SD) was hidden to improve the image's readability. **b**–**f** Progressive PAR reduction of αSyn RT-QuIC SAA reactions in different brain areas along the dilution series. The dilution coefficient is expressed in a logarithmic scale. Each point represents the average PAR value of positive quadruplicates at each dilution. Standard deviation (SD) was hidden to improve the image's readability. Linear regression lines were applied to values from patients in the same McKeith stage only when at least three values were available. *AP*, amygdala-predominant; *PAR*, protein aggregation rate; *RFU*, relative fluorescent units; *SAA*, seed amplification assay

To better characterize the relationship between the kinetic parameters of the αSyn RT-QuIC SAA and the number of αSyn seeds, we computed the protein aggregation rate (PAR) (1/lag phase), a measure of amyloid formation rate. In all cases, a progressive decrease in PAR along the dilution series (i.e., as the number of αSyn seeds in the sample decreased) was observed in each brain area (Fig. 2). In contrast, Imax and AUC did not show a regular decline over the dilution series, exhibiting an often irregular and unpredictable pattern in numerous brain areas (data not shown).

### Quantification of αSyn seeds in brain samples from αSyn LB+ patients

**Fig. 3:**
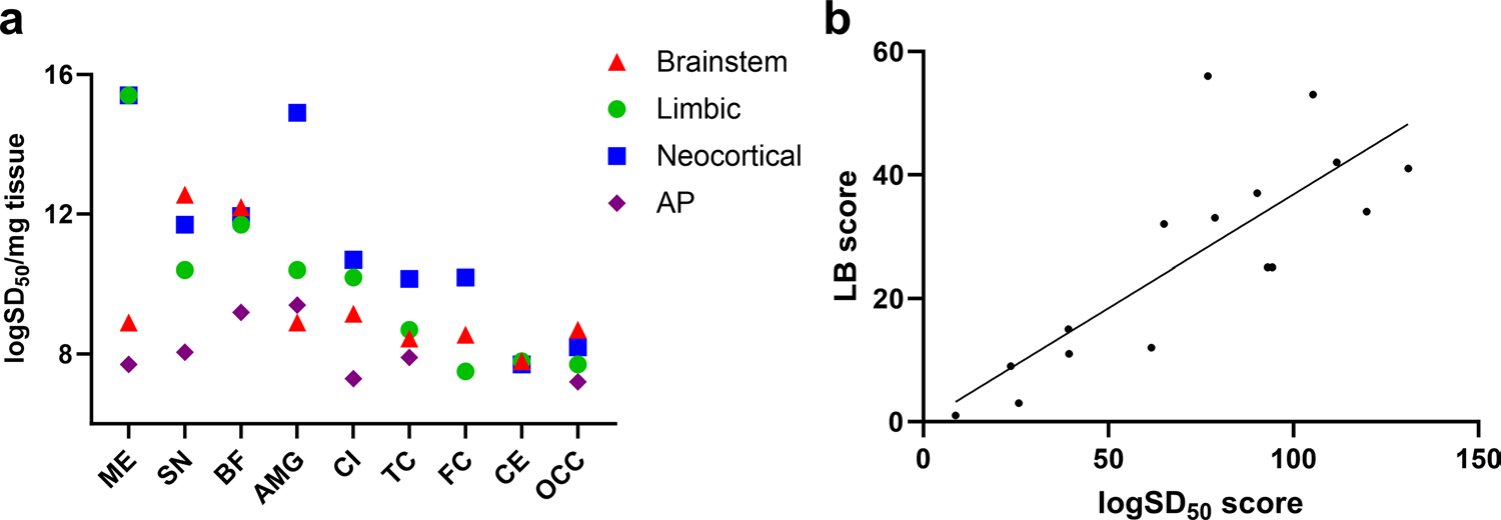
αSyn seeds quantification in brain samples **a** Average SD_50_/mg values in the main McKeith stages in different brain areas. Standard deviation (SD) was hidden to improve the readability of the image. **b** Correlation analysis between logSD_50_ score and LB score (*r* = 0.7903, 95% CI 0.4719 to 0.9263; *p* < 0.001). *AMG*, amygdala; *BF*, basal forebrain; *CI*, cingulate gyrus; *CE*, cerebellum; *FC*, frontal cortex; *LB*, Lewy body; *ME*, medulla oblongata; *OCC*, occipital cortex; *SN*, substantia nigra; *TC*, temporal cortex. SD_50_/mg values are expressed in logarithmic scale

We then estimated each area's average αSyn seed concentration using the Spearman-Kärber algorithm. We expressed it as logSD_50_ (seed concentrations giving 50% positive replicate reactions) per mg of tissue (Table 3). LogSD_50_/mg values were highly variable depending on the case and the brain area, ranging from 7.2 to 23.9, with the highest values detected in the substantia nigra, the basal forebrain, and the amygdala. When stratifying patients according to the McKeith stage, we reported no significant differences between the average seed concentrations of the four groups in the different brain areas (considering only the cases in which αSyn seeding activity was detected). However, the results showed a trend for the amygdala-predominant LBD cases to have lower average logSD_50_/mg values than the other groups in all brain areas where αSyn positivity was detected (medulla oblongata, substantia nigra, basal forebrain, cingulate gyrus, and temporal cortex), except in the amygdala, where the values were in the brainstem cases’ range (Fig. 3a).

**Table 3:** LogSD_50_/mg of brain homogenates and LB score results in different brain areas

Case		43	48	50	51	54	13	18	24	47	49	3	26	44	37	41	57
Braak stage		3	3	3	1	3	5	5	5	4	5	6	6	6			
McKeith stage		Brainstem					Limbic					Neocortical			AMG-predominant		
Brain areas																	
ME	SD_50_/mg	15.7	8.9	8.9	8.7	16.4	17.4	16.7	9.7	10.4	15.4	15.4	22.9	9.9	7.7	8.7	7.2
	IHC	6	3	4	1	6	6	6	6	6	6	6	6	6	3	Neg	Neg
SN	SD_50_/mg	11.4	14.7	7.9	Neg	13.7	23.9	20.2	8.9	9.2	10.4	11.7	20.9	9.7	7.2	8.9	Neg
	IHC	5	5	1	Neg	2	6	5	4	6	5	6	6	6	3	3	Neg
BF	SD_50_/mg	13.2	Neg	7.7	Neg	12.2	12.5	18.5	9.2	9.7	11.7	18.2	12.0	9.7	8,5	9.2	9.2
	IHC	6	Neg	1	Neg	6	6	6	6	6	6	6	6	4	Neg	3	Neg
AMG	SD_50_/mg	9.2	Neg	7.7	Neg	8.9	10.2	15.7	10.4	9.4	11.9	14.9	19.9	9.7	8.7	12.2	9.4
	IHC	Neg	Neg	Neg	Neg	Neg	3	2	4	4	6	5	2	6	6	5	3
CI	SD_50_/mg	9.7	Neg	7.2	Neg	8.7	9.2	12.7	10.2	11.2	8.9	10.7	18.2	9.9	7.2	7.4	Neg
	IHC	Neg	Neg	Neg	Neg	Neg	2	1	1	Neg	2	5	2	5	Neg	Neg	Neg
TC	SD_50_/mg	8.2	Neg	NA	NA	8.7	8.7	10.7	8.2	NA	8.7	9.4	10.9	NA	NA	7.9	Neg
	IHC	Neg	Neg	Neg	Neg	Neg	Neg	Neg	Neg	Neg	Neg	2	1	4	Neg	Neg	Neg
FC	SD_50_/mg	9.9	Neg	Neg	Neg	8.4	11.7	8.7	7.2	7.5	7.4	8.7	10.2	11.4	Neg	Neg	Neg
	IHC	Neg	Neg	Neg	Neg	Neg	Neg	Neg	Neg	Neg	Neg	1	1	4	Neg	Neg	Neg
CE	SD_50_/mg	7.2	Neg	NA	Neg	8.4	9.8	7.4	7.7	Neg	7.9	7.9	7.7	7.5	Neg	Neg	Neg
	IHC	Neg	Neg	Neg	Neg	Neg	Neg	Neg	Neg	Neg	Neg	Neg	Neg	Neg	Neg	Neg	Neg
OCC	SD_50_/mg	9.7	Neg	Neg	Neg	7.4	8.2	9.2	7.2	7.4	7.7	8.2	8.2	9.2	Neg	7.2	Neg
	IHC	Neg	Neg	Neg	Neg	Neg	Neg	Neg	Neg	Neg	Neg	Neg	Neg	Neg	Neg	Neg	Neg

*AMG*, amygdala; *BF*, basal forebrain; *CI*, cingulate gyrus; *CE*, cerebellum; *FC*, frontal cortex; *IHC*, immunohistochemical LB score; *ME*, medulla oblongata; *NA*, not available; *Neg*, negative; *OCC*, occipital cortex; *SN*, substantia nigra; *TC*, temporal cortex. SD_50_/mg values are expressed in logarithmic scale

To evaluate the association between the amount of LB pathology estimated through αSyn RT-QuIC SAA and that estimated through αSyn immunohistochemistry, we gave a combined logSD_50_ score to each case by summing the logSD_50_/mg values of all tested brain areas. LogSD_50_ score significantly correlated with LB score (*r* = 0.7903, 95% CI 0.4719–0.9263; *p* < 0.001) (Fig. 3b).

We also exploited αSyn seeds quantification analyses to compare the performance of αSyn RT-QuIC SAA and immunohistochemistry in detecting LB pathology. As previously mentioned, in 47/138 examined brain regions αSyn RT-QuIC SAA detected αSyn seeding activity notwithstanding a negative immunohistochemical assessment (Table 3). We found that all but 6 areas with logSD_50_/mg values ≤ 8.7 were negative at immunohistochemical evaluation, while only 12 above this threshold were negative.

### Differences in αSyn seeding activity in brain samples at SD_50_ dilution

Next, we tested the hypothesis that a different αSyn conformational strain would sustain the amygdala- predominant variant than that associated with the typical LBD stages. For this purpose, we looked for differences between αSyn seeding activity in the medulla oblongata and amygdala, assuming that an equal seed dose would be present in the sample at the dilution corresponding to the SD_50_. More specifically, we extrapolated the kinetic data (lag phase, Imax, and AUC) of the αSyn RT-QuIC SAA curves of the dilution related to the SD_50_. We compared the mean values across  the brainstem, limbic, neocortical, and amygdala-predominant stages and found no significant differences in αSyn RT-QuIC SAA kinetic parameters among the four groups in either the medulla oblongata or the amygdala (data not shown).

### Analysis of LBD prevalence in the CJD cohort

**Fig. 4:**
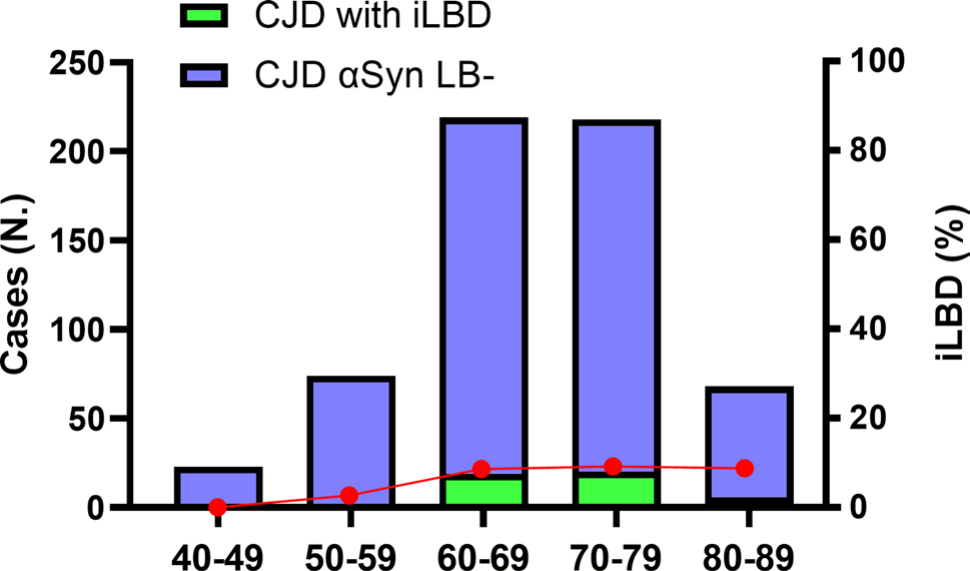
Age-specific iLBD prevalence rates in the CJD cohort. The green area shows the number of LB-positive cases at αSyn immunohistochemistry in each decade. The red line expresses these data in percentage. *iLBD*, incidental Lewy body disease, *CJD*, Creutzfeldt Jakob disease

In the last part of the study, we investigated the prevalence of iLBD in CJD patients, the largest and best-characterized subgroup of our NP cohort. Of the 604 examined CJD cases, 48 (7.9%) showed LB pathology. More in detail, 2 (0.3%) showed a neocortical, 19 (3.1%) a limbic, and 27 (4.5%) a brainstem stage (Table 1). Of note, no case exhibited an amygdala-predominant involvement. When stratifying patients by age, we found a prevalence of LBD in 3% of individuals aged 50–59, rising to 8–9% and staying stable in the following three decades (Fig. 4).

## Discussion

The performance against the gold standard, i.e., the neuropathologic diagnosis, represents a key issue for validating novel pathology-specific biomarkers. Studies of this kind are often limited by the long latency between the in vivo and postmortem assessments, especially in the case of usually long-standing pathological processes (e.g., AD or LBD). In this context, our collection of CSF-brain pairs from patients with rapidly progressive disease who died within a limited time frame after LP provides the best scenario for evaluating the specificity of an αSyn SAA assay to detect LB pathology. In the present study, we analyzed a comprehensive cohort of CSF samples and postmortem brain tissues, including a good representation of all LBD pathological stages.

Consistent with previous results [3, 7, 10, 21, 27, 55], we confirmed the assay's high overall specificity (98.6%) and sensitivity (81.4%) for LB pathology. Only three cases with a primary neuropathological diagnosis of Wernicke’s encephalopathy, AD, and sCJD yielded a positive test result without evidence of LB brain pathology. The finding of no positive αSyn seeding activity in brain homogenates from two of these cases (data not shown) excluded the hypothesis of prodromal situations in which the accumulation of misfolded αSyn preceded the appearance of LB pathology. The presence of LB pathology limited to the spinal cord and/or peripheral ganglia or a CSF contamination favoring the recombinant αSyn spontaneous aggregation may explain these incongruent positive results. Additional studies on larger sample cohorts are needed to address this critical issue further.

Regarding the assay sensitivity, there was a clear correlation with the LBD stage. The sensitivity was 100% in patients with neocortical or limbic LBD stages but decreased significantly in patients with the amygdala-predominant variant (50%) or a brainstem stage (60.9%), mainly when αSyn accumulation was sparse and limited to the medulla oblongata or the pons. Specifically, the sensitivity was 73.3% in those with Braak stage 3 and 37.5% in Braak stages 1 and 2. Of note, 13 of 14 patients in the brainstem stage with a negative αSyn SAA showed a low LB pathology load at the semiquantitative assessment. Furthermore, one exhibited an atypical LB pathology distribution showing sparse LBs and LNs in the medulla oblongata and substantia nigra skipping the pons.

To date, only a few studies in small cohorts have investigated the CSF αSyn SAA diagnostic accuracy in individuals with the amygdala-predominant form, while no detailed data are available for those with the brainstem stage of LBD [3, 21, 27]. Moreover, one of these studies [27] only analyzed postmortem ventricular CSF, which cannot be easily compared with the CSF obtained by LP at an early disease stage [57]. Fairfoul et al. reported that 31% (4/13) of AD brains with incidental LBD cases (with no details on the LBD stage) tested positive at CSF αSyn SAA [21]. More recently, Hall et al. detected αSyn seeding activity in the postmortem ventricular CSF of 0% (0/2) individuals with “brainstem only”, 71% (5/7) with “amygdala only”, and 40% (2/5) of those with “LB pathology restricted to amygdala and brainstem” [27]. Finally, Arnold et al. reported a low (3/21, 14.3%) αSyn SAA sensitivity using antemortem CSF in the amygdala-predominant LBD variant [3].

There is no definite explanation for the reduced sensitivity of the αSyn SAA in the brainstem and amygdala-predominant stages. Hypothetically, this could be due to either the lower brain LB pathology loads compared to full-blown LBD in limbic and neocortical stages or, especially for the amygdala-predominant variant, the presence of a distinct pathological αSyn conformer, exhibiting no aggregation at all or a different kinetic profile compared to the αSyn associated with typical LBD. Our demonstration of a clear threshold effect, with the assay yielding negative results in all but four cases with a LB score < 10 out of 78, and the evidence of a significant correlation between the number of positive replicates and the LB score strongly support the hypothesis of an association between the assay sensitivity and the overall brain LB pathology load.

The data we obtained by performing the αSyn SAA in brain homogenates from different regions further supports the positive association between LB pathology burden and assay sensitivity. First, we demonstrated for the first time a robust positive correlation between the brain αSyn seeding activity and the LB pathology load evaluated by αSyn immunostaining, thus excluding that a significant proportion of the αSyn aggregates forming LBs and LNs may lack seeding properties. Second, in each examined brain area showing LB pathology, we did not find significant differences in the mean logSD_50_/mg values among brains stratified according to the McKeith stage, thus excluding the hypothesis of misfolded αSyn isoforms contributing differently to the overall seeding activity depending on the LBD stage. As the only possible exception, we found a trend toward lower average logSD_50_/mg values for the amygdala-predominant cases compared to those with typical LBD. However, although the small sample size does not allow us to draw firm conclusions, we did not find differences in amygdala seeding activity between the amygdala-predominant cases and those in other stages. Further studies should investigate the structural, physicochemical, and seeding properties of αSyn in this peculiar histopathological variant compared to typical LBD.

Our results, combined with those of previous studies [3, 27], indicate that the αSyn SAA have enough sensitivity to detect all symptomatic patients with DLB or PD related to LB pathology. Indeed, DLB patients and most of those with PD are expected to have reached the limbic LBD stage already at clinical onset. Moreover, in PD patients, a high LB pathology load in the brainstem is expected to explain the significant neurodegeneration in the substantia nigra. However, CSF αSyn SAAs have shown high diagnostic performance in confirming LB pathology not only in DLB and PD patients but also in most of those affected by prodromal LBD syndromes, such as Isolated Rapid Eye Movement Sleep Behavior Disorder (iRBD) or mild cognitive impairment due to LBD (MCI-LB) and even in asymptomatic individuals [15, 32, 54, 55]. In this context, our finding of an incomplete sensitivity of the αSyn SAA for the brainstem stage of LBD might be interpreted as partially diverging from the results of the above clinical studies and deserves specific comment. The issue mainly concerns the asymptomatic individuals and those with iRBD since the 90–95% sensitivity demonstrated for MCI-LB by our SAA in the clinical setting [54] is entirely in line with the present results given that the LBD Braak stage expected in these patients is > 3 [33]. Regarding iRBD, we recently demonstrated a 75% sensitivity of our CSF αSyn SAA in the largest clinical cohort tested to date [32]. The three other studies with significant cohorts of iRBD patients conducted to date showed 90% [31] and 84–94% [15, 58] assay sensitivities. Possible explanations for these differences are the different SAA protocols, including the recombinant protein and differences in patients’ characteristics. Regarding the latter, it must be emphasized that iRBD patients often manifested other signs of “prodromal” LBD, including MCI and motor deficits, suggesting a relatively high LB pathology burden and spread, thus justifying, in line with our current results, a higher than expected sensitivity of the SAA assay. For example, the percentage of patients with iRBD with coexistent MCI or motor signs was significantly higher in the study reporting an αSyn SAA sensitivity of 90% compared to ours in which the sensitivity was 75%. In summary, the results of the present study, in line with recent imaging findings [35], strongly suggest that iRBD patients harbor, on average, a Braak stage 3 of LB pathology, with significant variations from case to case mainly depending on the prodromal LBD score determined by the associated neurological signs. Finally, our recent finding of 8% αSyn SAA positivity in a large cohort of cognitively unimpaired individuals [47] suggests that even the asymptomatic “incidental” LBD status may be associated with a variable LB pathology load, likely including Braak stages 3 and 4. The fact that all αSyn SAA-positive patients with CJD or a non-degenerative etiology included in the present study did not have any anamnestic evidence of motor signs or cognitive decline before the onset of a rapidly progressive disease and yet showed LB pathology up to Braak stage 4 supports this conclusion.

In brain sample experiments, we sought to analyze how the kinetic parameters of the αSyn SAA fluorescence signal vary depending on the number of αSyn seeds in the sample. For this purpose, we extrapolated each run's Imax, AUC, and lag times and studied their values’ distribution along the dilution series. We computed the PAR (1/lag time) as a measure of the amyloid formation rate to deal with negative reactions (which show a lag phase virtually approaching infinity so that the rate of amyloid formation can be assumed equal to 0), as reported [30]. Imax and AUC did not show a regular decline over the dilution series, exhibiting an unpredictable pattern, likely depending not only on the amount of seed but also heavily on the experimental conditions. Conversely, following the amyloid seeding model, which predicts a linear relationship between the initial seed concentration and the rate of amyloid formation [30, 68], we found a linear dependence of the PAR (and thus the lag phase) on seed concentration. This paves the way for using the lag phase as a surrogate for the number of seeds in the sample. These results have significant implications for evaluating and validating the reliability of the “quantitative” information provided by the αSyn SAA. Developing a quantitative assay will allow the evaluation of disease severity and progression and may take the αSyn SAA to the next level. Preliminary evidence indicates that the assay might provide quantitative data indirectly revealing the number of protein seeds in the biosample and the associated LB pathology burden in addition to the well-established reliable qualitative dichotomous (positive/negative) response. The data we obtained in the present study indicate that both the number of positive replicates and the lag phase are closely related to the αSyn burden in the CNS and likely represent the two most reliable variables to pursue the clinical validation of a quantitative αSyn SAA.

As one of the main findings, we demonstrated that in brain tissue αSyn SAA detect misfolded αSyn with higher sensitivity than immunohistochemistry. Additionally, we showed a strong correlation between the overall seed quantity, estimated through the logSD_50_/mg score and the brain LB pathology load, assessed through immunohistochemistry. The formation of LBs and LNs is likely a multistep process starting from monomeric misfolded proteins and involving intermediate soluble species (i.e., oligomers and protofibrils), which further aggregate in insoluble fibrils, eventually forming immunohistochemically detectable deposits [60]. Our results suggest that seeds triggering αSyn SAA reactions likely include pre-fibrillary aggregates. The finding supports the numerous pieces of evidence suggesting that pre-fibrillary species are those mediating αSyn pathology spread and toxic effects [8]. In this perspective, it is possible that αSyn SAA may not only have diagnostic applications but also a potential role in monitoring the response to therapy in case future treatments targeting the early stages of the aggregation process will be available.

In the last part of the study, we reported the data obtained in our cohort of CJD patients screened for the presence of LB pathology. Consistent with previous evidence [25], we found that almost 8% of CJD patients showed LB pathology (with 4.5% cases in the brainstem, 3.1% in limbic, 0.3% in neocortical stages, and none in the amygdala-predominant variant). Based on the assumption that there is no association between prion disease and LBD, which is compatible with the current literature, we speculate that the prevalence of LB pathology in our wide case series of consecutive well-characterized CJD patients lacking clinical evidence of cognitive decline and/or motor disturbances before the rapid onset of symptoms related to CJD, can be bona fide considered an estimate of the frequency of iLBD in the elderly population. Previous studies estimated iLBD prevalence ranging between 8 and 15% in neurologically unimpaired individuals [9, 11, 22, 34, 42, 44, 51, 52, 64, 66]. In line with our results, in most cases, iLBD follows the Braak progression model [33], although it can sometimes be limited to limbic or neocortical areas [22, 33]. In our cohort, iLBD prevalence increased significantly in the transition between the sixth and seventh decades, consistent with previous results [52, 66]. However, at variance with previous studies [52, 66], which reported a steep and progressive increase from the sixth decade onwards (reaching up to 15–18% of cognitively normal subjects > 90 years old), iLBD prevalence in our cohort remained substantially stable across decades (around 8–9%). Interestingly, similar results have been obtained in recent studies employing CSF αSyn SAA to estimate the prevalence of iLBD in cognitively unimpaired individuals [15, 47, 56]. Lastly, although amygdala LB pathology has been reported to occur frequently in concomitance with other protein misfolding disorders (e.g., AD and other tauopathies [46, 62]), we found no cases of the amygdala-predominant LBD variant in our CJD cohort, not even in sCJD subtypes with significant pathological involvement of the amygdala (i.e., VV2, VV1, and MV2K) [5, 6], supporting the prominent role of AD-related tau pathology in determining this subtype of focal LBD pathology.

The main study limitation is the low number of amygdala-predominant and Braak stage 1/2 cases. Furthermore, we are aware that the results on brain homogenates are strongly conditioned by the sampling accuracy, with some areas more accessible than others and harboring more widespread pathology. However, we are confident that including many areas from a relatively large number of subjects at each stage has minimized the inherent variability associated with the sampling procedure. We are also aware that our cohort mainly includes patients who, in most cases, do not have LBD as their primary diagnosis, so we could not correlate the assay results with clinical variables (e.g., motor or cognitive scores). As a further limitation, our cohort comprised mostly patients with a rapidly progressive disease course, which therefore may not be representative of the overall population affected by LB pathology. Finally, the inclusion, albeit in a small number, in the αSyn LB+ cohort of cases with a timespan LP-death > 1 year is a further limitation of the study.

In conclusion, taking advantage of a large neuropathologic cohort including LBD cases in various stages, our results confirm the virtually full accuracy of our ante mortem CSF αSyn SAA as a marker of LB pathology in limbic and neocortical stages, making it a revolutionary tool for LBD diagnosis. However, we observed lower αSyn SAA sensitivity in patients in the early stages (especially in cases of pathology limited to the medulla and/or pons) or with focal pathology (e.g., amygdala-predominant variant). Together with the CSF data, the results obtained by performing αSyn SAA on brain homogenates from different regions suggest that the LB pathology burden is the factor that likely mostly affects the assay sensitivity. Furthermore, kinetic analysis of the fluorescence signal along the dilution series of brain samples suggests that αSyn SAA can provide "quantitative" information, with the lag phase and the number of positive replicates being the most promising and reliable variables to develop a “quantitative” αSyn SAA prospectively. Finally, taking advantage of our well-characterized CJD cohort, we estimated the prevalence of iLBD in the elderly population to be about 8%, thus emphasizing the importance of developing a reliable method for early and accurate LBD diagnosis.

Future efforts should focus on improving the assay sensitivity in cases with low LB pathology load, standardizing the protocol across laboratories, evaluating the impact of its inclusion in PD and DLB diagnostic criteria, and fully developing its “quantitative” potential.

### Supplementary Information

Below is the link to the electronic supplementary material.

Supplementary file1 (DOCX 107 kb)

## Data Availability

The datasets used and analyzed during the current study are available from the corresponding author upon reasonable request.
