# Supplementary material for: Performance of a seed amplification assay for misfolded alpha-synuclein in cerebrospinal fluid and brain tissue in relation to Lewy body disease stage and pathology burden
Source: Acta Neuropathol. 2024 Jan 19;147(1):18. doi: 10.1007/s00401-023-02663-0 (PMC10799141; doi:10.1007/s00401-023-02663-0)
Supplement: Supplementary file 1 — Supplementary file1 (DOCX 107 kb) [file 401_2023_2663_MOESM1_ESM.docx]

**Performance of a seed amplification assay for misfolded alpha-synuclein in cerebrospinal fluid and brain tissue in relation to Lewy body disease stage and pathology burden**

Giuseppe Mario Bentivenga^1^, Angela Mammana^2^, Simone Baiardi^1^, Marcello Rossi^2^, Alice Ticca^1^, Franco Magliocchetti^2^, Andrea Mastrangelo^1^, Anna Poleggi^3^, Anna Ladogana^3^, Sabina Capellari^1,2^, Piero Parchi^1,2^

Giuseppe Mario Bentivenga and Angela Mammana have contributed equally to the study.

^1^ Department of Biomedical and Neuromotor Sciences, University of Bologna, Bologna, Italy

^2^ IRCCS, Istituto Delle Scienze Neurologiche di Bologna, Bologna, Italy

^3^ Department of Neurosciences, Istituto Superiore di Sanità, Rome, Italy

Correspondence to Piero Parchi:

Email: piero.parchi@unibo.it

Supplementary Table 1 – Pathological entities included in the LB αSyn+ CSF cohort and CSF RT-QuiC results

| **Case** | **Sex** | **Age at death** | **Age at LP** | **Main NP diagnosis** | **Secondary NP diagnosis** | **CSF αSyn RT-QuIC SAA results** | **McKeith stage** | **Braak stage** | **LB score (0-78)** |
| --- | --- | --- | --- | --- | --- | --- | --- | --- | --- |
| 1 | F | 79 | 79 | LBD | AD low | Positive | Neocortical | 6 | 46 |
| 2 | F | 83 | 83 | LBD | AD intermediate | Positive | Neocortical | 6 | 56 |
| 3 | F | 81 | 81 | LBD | AD low | Positive | Neocortical | 6 | 53 |
| 4 | M | 74 | 74 | sCJD MM1 | AD low, LBD | Positive | Limbic | 4 | 33 |
| 5 | F | 83 | 83 | sCJD MM1+2C | AD low, LBD | Positive | Limbic | 4 | 24 |
| 6 | F | 81 | 81 | LBD | AD intermediate | Positive | Neocortical | 6 | 54 |
| 7 | M | 80 | 80 | LBD | AD intermediate | Positive | Neocortical | 6 | 52 |
| 8 | F | 77 | 76 | LBD | AD intermediate | Positive | Neocortical | 6 | 39 |
| 9 | M | 74 | 74 | LBD | AD low | Positive | Neocortical | 6 | 52 |
| 10 | F | 61 | 60 | sCJD MM1+2C | AD low, LBD | Negative | Brainstem | 3 | 10 |
| 11 | F | 75 | 75 | sCJD MM1 | AD low, LBD | Positive | Limbic | 5 | 43 |
| 12 | M | 67 | 67 | gCJD V210I-129M | LBD | Positive | Limbic | 4 | 19 |
| 13 | M | 72 | 71 | LBD | AD low | Positive | Limbic | 5 | 42 |
| 14 | M | 81 | 80 | AD high | LBD | Negative | Amygdala | 0 | 9 |
| 15 | M | 75 | 75 | sCJD MM1 | AD low, LBD | Positive | Neocortical | 6 | 47 |
| 16 | F | 81 | 79 | LBD | AD intermediate | Positive | Neocortical | 6 | 56 |
| 17 | F | 60 | 60 | Metabolic encephalopathy | LBD | Negative | Brainstem | 2 | 7 |
| 18 | M | 73 | 73 | LBD | AD low | Positive | Limbic | 5 | 34 |
| 19 | M | 69 | 69 | sCJD MM1 | AD low, LBD | Negative | Brainstem | 3 | 18 |
| 20 | M | 60 | 60 | sCJD MM1+2C | AD low, LBD | Positive | Limbic | 5 | 31 |
| 21 | M | 63 | 61 | gCJD R208H-129M | LBD | Positive | Brainstem | 3 | 19 |
| 22 | F | 79 | 79 | sCJD VV2 | AD low, LBD | Positive | Brainstem | 3 | 17 |
| 23 | M | 77 | 77 | LBD | AD intermediate | Positive | Limbic | 5 | 37 |
| 24 | F | 76 | 75 | Primary CNS lymphoma | LBD | Positive | Limbic | 5 | 33 |
| 25 | M | 67 | 67 | sCJD MM1 | LBD | Positive | Limbic | 5 | 33 |
| 26 | M | 77 | 77 | LBD | AD low | Positive | Neocortical | 6 | 41 |
| 27 | M | 65 | 64 | LBD | AD high | Positive | Neocortical | 6 | 45 |
| 28 | M | 87 | 87 | AD low | LBD | Positive | Brainstem | 3 | 15 |
| 29 | M | 76 | 76 | sCJD MM1 | AD low, LBD | Positive | Brainstem | 3 | 27 |
| 30 | M | 81 | 81 | sCJD MV2K+2C | AD low, LBD | Negative | Brainstem | 2 | 7 |
| 31 | F | 84 | 84 | LBD | subcortical arteriosclerotic encephalopathy | Positive | Limbic | 5 | 45 |
| 32 | M | 73 | 73 | LBD | AD intermediate | Positive | Neocortical | 6 | 45 |
| 33 | F | 65 | 65 | sCJD MM1 | LBD | Positive | Brainstem | 2 | 5 |
| 34 | M | 83 | 83 | LBD | AD intermediate | Positive | Neocortical | 6 | 56 |
| 35 | M | 75 | 70 | LBD | AD high | Positive | Limbic | 5 | 37 |
| 36 | M | 73 | 72 | sCJD VV2 | AD low, LBD | Negative | Brainstem | 2 | 9 |
| 37 | M | 83 | 83 | LBD | AD high | Positive | Amygdala | 0 | 15 |
| 38 | M | 62 | 62 | sCJD MV1 | AD low, LBD | Positive | Brainstem | 1 | 7 |
| 39 | F | 66 | 66 | sCJD MM1+2C | AD low, LBD | Positive | Brainstem | 1 | 2 |
| 40 | F | 73 | 73 | sCJD VV2 | AD intermediate, LBD | Positive | Limbic | 5 | 42 |
| 41 | M | 70 | 70 | LBD | AD high | Positive | Amygdala | 0 | 12 |
| 42 | M | 56 | 56 | gCJD V210I-129M | AD low | Negative | Brainstem | 3 | 3 |
| 43 | M | 75 | 75 | LBD | AD low | Positive | Brainstem | 3 | 25 |
| 44 | F | 79 | 78 | LBD | AD intermediate | Positive | Neocortical | 6 | 56 |
| 45 | F | 63 | 63 | sCJD MM2C+1 | AD low | Negative | Brainstem | 3 ^a^ | 7 |
| 46 | F | 62 | 61 | sCJD MM1 | LBD | Positive | Limbic | 4 | 28 |
| 47 | F | 82 | 82 | Primary CNS lymphoma | AD low, LBD | Positive | Limbic | 4 | 32 |
| 48 | F | 80 | 80 | LBD | AD intermediate | Positive | Brainstem | 3 | 9 |
| 49 | F | 76 | 76 | sCJD MM1 | AD intermediate, LBD | Positive | Limbic | 5 | 37 |
| 50 | M | 76 | 76 | Subcortical arteriosclerotic encephalopathy | AD low, LBD | Positive | Brainstem | 3 | 11 |
| 51 | M | 75 | 75 | Intravascular lymphoma | AD low, LBD | Negative | Brainstem | 1 | 1 |
| 52 | F | 75 | 72 | AD intermediate | LBD | Positive | Neocortical | 6 | 53 |
| 53 | M | 62 | 62 | sCJD MM1 | AD low, LBD | Positive | Limbic | 5 | 32 |
| 54 | M | 84 | 84 | AD low | LBD | Positive | Brainstem | 3 | 25 |
| 55 | M | 64 | 64 | sCJD MM1 | LBD | Positive | Brainstem | 3 | 21 |
| 56 | F | 67 | 67 | sCJD MM1 | AD low, LBD | Positive | Brainstem | 3 | 15 |
| 57 | M | 57 | 52 | FTLD-TDP43 | AD low, LBD | Negative | Amygdala | 0 | 3 |
| 58 | M | 72 | 72 | sCJD MM1+2C | AD low, LBD | Positive | Brainstem | 3 | 27 |
| 59 | F | 63 | 63 | sCJD MV1 | AD low, LBD | Negative | Brainstem | 1 | 4 |

Abbreviations: AD, Alzheimer’s disease; CNS, central nervous system; CSF, cerebrospinal fluid; FTLD, frontotemporal lobar degeneration; gCJD, genetic Creutzfeldt-Jakob disease; LBD, Lewy body disease; LP, lumbar puncture; SAA, seed amplification assay; sCJD, sporadic Creutzfeldt-Jakob disease

^a^atypical LBD distribution: sparse LBs and LNs in the medulla oblongata and substantia nigra skipping the pons


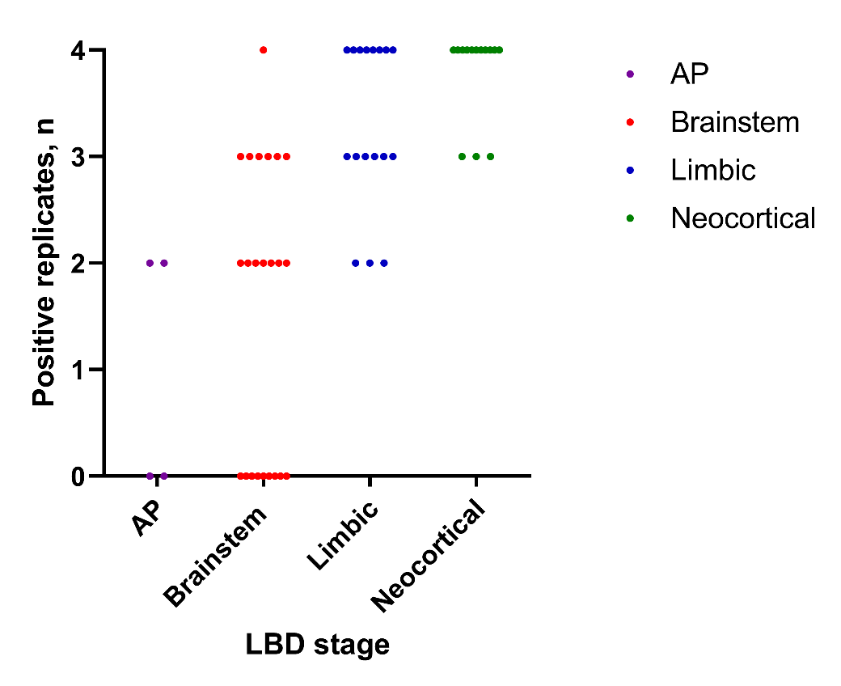


Supplementary Fig. 1 – CSF αSyn RT-QuIC SAA results (number of positive quadruplicates) in αSyn LB+ patients according to LBD stage. The number of positive replicates showed a tendency to increase in more advanced LBD stages.

AP, amygdala-predominant; LBD, Lewy body disease
